# Community health worker interventions to improve access to health care services for older adults from ethnic minorities: a systematic review

**DOI:** 10.1186/s12913-014-0497-1

**Published:** 2014-11-13

**Authors:** Ilona Verhagen, Bas Steunenberg, Niek J de Wit, Wynand JG Ros

**Affiliations:** Julius Center for Health Sciences and Primary Care, University Medical Center Utrecht, Mailbox 85500, 3508 GA Utrecht, The Netherlands

**Keywords:** Community health worker, Ethnic minority older adults, Access to health care, Systematic review

## Abstract

**Background:**

The health status of older adults belonging to ethnic minorities in Western countries is an important public issue because their health is often less favourable than that of older adults from the majority population. In addition, the number of older adults belonging to ethnic minorities is increasing rapidly in Western countries. The introduction of community health workers (CHWs) has proven to be successful in addressing health disparities among ethnic minorities; however, an overview of CHW’s benefits for older adults is absent in the literature. We reviewed the literature to explore whether CHWs are also effective in improving the health and the delivery of health care services to ethnic minority older adults in Western countries.

**Methods:**

We searched the PubMed database (2002-Present) for RCTs published on the use of CHWs in Western countries.

**Results:**

Out of the 729 studies identified, seven studies met our inclusion criteria. The effectiveness of the implementation of CHW programmes in older adults belonging to ethnic minorities is not univocal. In two studies, we found no significant differences. In five studies, we found some positive effects. We did not find negative effects in any of the studies. For better interpretation of the results, effect ratios (ERs) were calculated as the number of positive findings divided by the total number of measured findings. Substantial effects on the access to care (mean ER = 0.58) and on health behaviour (mean ER = 0.45) were found. The mean ER for health outcomes was considerably lower (mean ER = 0.17).

**Conclusion:**

We found indications that CHWs serve as a means of improving health care use and health behaviour and, to a lesser extent, health outcomes among ethnic minority older adults. Further research is required to draw more solid conclusions on the effectiveness of CHW interventions in this target group. This is particularly important for Western countries in which the number of ethnic minority older adults has increased significantly because their health status is mostly unfavourable and their access to health care services is often limited.

**Electronic supplementary material:**

The online version of this article (doi:10.1186/s12913-014-0497-1) contains supplementary material, which is available to authorized users.

## Background

The health status of most ethnic minority groups in Western countries is poorer than the health status of the majority population [1-5]. This is especially applicable for ethnic minority older adults. Limited access to health care services has been reported to be an important factor for these disparities in health [6,7] and is in part caused by limited knowledge about health care facilities, language problems, and financial barriers. Intercultural differences in the perception of health needs and reasons for consultation may be other important contributing factors [8,9].

Culturally sensitive interventions such as the introduction of community health workers (CHWs) have been used to address health disparities among ethnic minorities. CHWs share the same ethnic background, speak the same language, are aware of the health beliefs and understand the barriers to health care that ethnic minority adults experience. In addition, they act as intermediaries between community members and providers of health care services [10,11]. In practice, CHWs are employed in various programmes to reduce disparities in health, most frequently related to specific health conditions such as cancer, diabetes, hypertension, asthma, nutrition, and tobacco control [12]. CHWs work in various functions: to improve health knowledge, to increase access to health care, to induce behavioural changes, and to reduce health care costs [13].

In the US, the implementation of CHWs has been identified as a strategy to address disparities in health status among ethnic minorities since the 1960s. Over the last decades, the number of intervention studies aiming to address health disparities using CHWs has increased rapidly in the US. The effectiveness and content of CHW interventions for ethnic minorities have been reported in a previous review of ethnic minority women of all ages in the US [14]. CHWs have been demonstrated to be effective in increasing health knowledge, changing health behaviour, and increasing access to care in this target group. However, an overview of CHW benefits for older adults is lacking in the literature.

We aimed to investigate whether CHWs are also effective in providing the aforementioned benefits to ethnic minority older adults. The number of older adults from ethnic minorities is increasing rapidly in Western countries, and their utilisation of health facilities has been reported to lag behind [5]. Better access to health care would optimising their health and enable them to maintain independence, improve societal participation, and diminish existing disparities. Therefore, we systematically reviewed the literature on the implementation of CHW programmes in ethnic minority older adults in Western countries.

## Methods

### Protocol and registration

Methods of analysis and inclusion were specified in advance, but not documented in a review protocol.

### Search strategy

We searched the PubMed database (2002-Present) for studies on the use of CHWs. The last search was run on 25 October 2012. We used the search term “community health workers” and 47 related terms applied to CHWs in various health-promotion programmes as described in a review by Andrews [14]. For our review, we added the terms “cultural brokers” and “health brokers” to the search, resulting in the following search terms (both singular and plural): “auxiliary health workers”, “canvassers”, “community health advisors”, “community health advocates”, “community health aides”, “community health representatives”, “community health workers”, “community helpers”, “community workers in human services”, “consejeras”, “cultural brokers”, “family health counsellors”, “family health promoters”, “health aides”, “health assistants”, “health brokers”, “health education aides”, “health care expediters”, “health facilitators”, “health guides”, “health hostesses”, “health liaisons”, “health outreach workers”, “health promoters”, “indigenous environmental workers”, “indigenous health aides”, “indigenous health professionals”, “indigenous lay workers”, “indigenous workers”, “informal helpers”, “lay community health workers”, “lay health advisors”, “lay volunteers”, “lay workers”, “natural caregivers”, “natural helpers”, “navigators”, “neighbourhood-based public health workers”, “neighbourhood representatives”, “neighbourhood workers”, “nonprofessional workers”, “outreach workers”, “peer counsellors”, “peer educators”, “promotoros”, “public health aides”, “paraprofessionals”, “raidat rifiat”, “resource mothers”, “volunteer health educators”.

In addition, we used the PubMed filters “full text available”, “English language”, “published in the last ten years”, and “age 45 years and over”.

### Inclusion criteria

- Type of intervention: CHW interventions focussed on health-related outcomes.
- Type of studies: randomised controlled trials (RCTs) that compared a CHW arm against a control arm receiving either usual care, no intervention, or another intervention.
- Target population: ethnic minority older adults (50 years and over). Studies in which ethnic minority older adults were at least 70% of the total sample or in which the results were specified for ethnic older adults. The minimum age was set to 50 because adults belonging to ethnic minority groups often experience health problems at a younger age than the majority population [15].
- Type of CHW: paid or voluntary, carried out functions associated with health care delivery, involved in reaching and serving hard-to-reach and underserved ethnic minorities, not a formally trained health care professional, and not an extender of the formal health care system.
- Outcomes: outcomes related to health and delivery of health care services.
- Setting: studies conducted in Western countries. Western countries were limited to Europe, the US, Canada, Australia, and New Zealand because these countries currently host a growing number of aging adults belonging to ethnic minorities.
- Time period: studies published in the last ten years. The time period was limited to the last ten years to include recently conducted studies.

### Exclusion criteria

Studies were excluded if:

- The intervention was not adequately described to determine that it was a CHW intervention.
- The effects were not properly described to determine whether the CHWs produced the effects.

### Methodological quality

Two reviewers (IV and BS) independently performed the methodological quality assessment of the included studies. Because all studies were RCTs, the methodological quality was assessed using the Cochrane criteria for RCTs from the Dutch Cochrane Centre [16] (Table 1). Two items (blinding of participant and blinding of care provider) were disregarded because these items were not applicable to the evaluated field interventions. Two items were added (statistical power and validity and reliability of the measuring instruments). Follow-up was considered acceptable if the loss to follow-up did not exceed 20% because the extant research suggests that more than 20% loss poses serious threats to validity [17].

**Table 1:** **Methodological quality of included studies** ^**1**^

**RCTs**	**1. Randomisation**	**2. Concealment of allocation**	**3. Blinding of outcome assessors**	**4. Comparability of groups**	**5. Loss to follow-up**	**6. Intention-to-treat analysis**	**7. Comparability of treatment**	**8. Power analysis**	**9. Objective and validated measuring instruments**	**Total score**
Hunter et al. [22]	+/−	?	?	+	+	+	+	-	+/−	5.0
Jandorf et al. [21]	+/−	?	-	+	+	+	+	-	+	5.5
Gary et al. [24]	+	+	+	+	+	-	+	-	+	7.0
Balcázar et al. [23]	+/−	+	-	+/−	+	+	+	-	+	5.5
Maxwell et al. [20]	+	-	NA^2^	+	+	+	+	+	+/−	7.3
Hayashi et al. [19]	+/−	-	+/−	+	+/−	-	+/−	-	+	4.0
Coleman et al. [18]	+/−	-	NA^2^	+	+/−	-	+	-	+/−	3.9

^1^The methodological quality was assessed using the Cochrane criteria for RCTs from the Dutch Cochrane Centre [16].

^2^NA = not applicable to the intervention.

Each item scored 1 point (+) if the criterion was met, 0.5 points (+/−) if the criterion was partially met, and 0 points (−) if the criterion was not met. The score was “?” if the information was not reported or unclear. We reported “NA” if the item was not applicable. The total score for the methodological quality was calculated by summing the subscores. We adjusted the total scores for “NA” (by summing the subscores, dividing this score by the total number of applicable items, and multiplying it by the total number of items). Methodological quality was considered “high” between 8 and 9, “moderate” between 4 and 7, and “low” between 1 and 3.

### Data extraction and analysis

Two reviewers (IV and BS) independently screened the importance of all titles and abstracts searched in PubMed. The full text of all articles that were considered as possibly relevant was further screened based on inclusion criteria by either reviewer. Selected articles eligible for inclusion were compared, and discrepancies were discussed by the reviewers and resolved by consensus. When the two reviewers disagreed on the eligibly, the decision was referred to a third reviewer (WR).

In addition, one reviewer (IV) extracted data from the included studies and documented these data on a data extraction sheet. Information was extracted from each included study on: (1) study features (including objective, setting, study design, length of follow-up, health focus); (2) number of study participants; (3) type of intervention; (4) type of CHW (including term used for the CHW, CHW’s role, paid or volunteer, number of CHWs, training, supervision); (5) characteristics of the target population (including age, gender, ethnicity/race); (6) results. If the effect of CHW interventions was evaluated on more than one outcome (e.g., health behaviour and health outcomes), the impact on each outcome was evaluated and documented independent of the results for the other outcomes.

The studies were categorised according to CHW roles and outcomes assessed. According to Andrews [14], the CHW roles were grouped into four categories based on the aim of their intervention: outreach, case management, data collection, and education. In line with other reviews on CHWs, outcomes were grouped into three categories: access to care, health behaviour, and health status.

The *p*-values were grouped into three categories: *p* ≤ 0.05 was considered “significant”, *p* > 0.05 and ≤0.10 as “a trend towards significance”, and *p* > 0.10 as “not significant”.

Because the studies differed in the number of measured outcomes, we assessed the “effect ratio (ER)” by dividing the total number of (trend towards) significant outcomes by the total number of measured outcomes. The ER made comparisons between studies possible because it took the differences in the number of measured outcomes into account. The ER ranged from 0 to 1. A score of 0 indicated that there was no significant effect, and a score of 1 indicated that all measured outcomes were significant. We calculated the mean ER per outcome category (access to care, health behaviour, and health outcomes) by summing the ERs per outcome category and dividing the total score by the total number of ERs for that particular category.

Because one study [18] did not report the significance of between-groups differences, we considered the CHW arm and the usual care arm as significantly different if the confidence intervals did not overlap.

## Results

**Figure 1:**
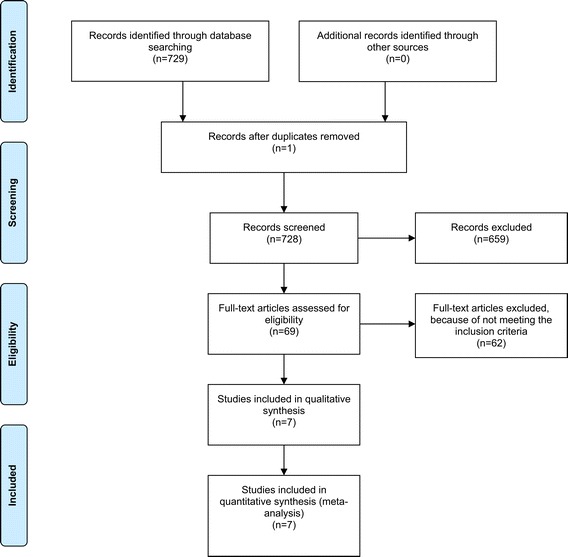
**Flow diagram.**

As a result of our selection process, 729 studies were identified in PubMed. After an abstract review, 73 studies were considered potentially eligible for inclusion. Eight studies could not be retrieved online, and the full text of four of them was obtained by contacting the first author. Of the resulting 69 studies, 62 were excluded for not meeting the inclusion criteria (Figure 1). The resulting seven studies were included in this review. Two papers were based on data from the same study population [18,19]. We evaluated the content of these papers as two separate studies because of the difference in their study objectives.

All included studies took place in the US and were focussed on specific ethnic populations (Additional file 1: Table S1). Five studies targeted Hispanics/Latinos, one study targeted African Americans, and one study targeted Koreans. In four studies, the intervention was solely focussed on women. Additionally, in four studies, CHWs had more than one role. CHWs had an outreach role in which they recruited participants for the study (n = 1). In four studies, CHWs were case managers by e.g., offering emotional support, scheduling appointments, providing reminders of appointments, providing educational information, arranging travel, and accompanying patients to appointments. In three studies, CHWs collected data, and in five studies, they provided educational programmes.

### Methodological quality

As shown in Table 1, two studies reported concealment of the allocation. Six studies did not use a power analysis to calculate the sample size. In four studies, an intention-to-treat analysis was conducted. Five studies reported an acceptable percentage of loss to follow-up (not greater than 20%). Two studies met this criterion to some extent because loss to follow-up exceeded the 20% in the intervention arm (22%) but not in the usual care arm (20%). Six studies reported the comparability of groups. One study fulfilled this criterion partially because not all baseline characteristics were identical. In four studies, objective and validated measuring instruments (laboratory measurements and/or questionnaires) were used. Six studies reported the comparability of treatment (except the introduction of CHWs). One study met this criterion partially because those in the CHW arm were referred to educational classes at three of the four sites, while at one site, verbal education was provided.

The total scores (within a range of 0–9 points) of the methodological quality ranged from 3.9 to 7.3. Six studies scored four points or more, indicating a moderate methodological quality. None of the studies had an optimal methodological quality.

### Effectiveness of interventions

The outcomes of the studies were reported in three different categories: access to care, health behaviour, and health status (Additional file 1: Table S1). No studies with knowledge as an outcome were found. Three studies evaluated more than one outcome category.

### Access to care

A study of Korean women showed positive results. Using a full case analysis, statistically significant (*p* = 0.001) higher rates of completion of diagnostic follow-up after breast cancer screenings were reported in the CHW intervention arm compared to the usual care arm [20]. An intention-to-treat analysis showed a trend towards significance (*p* = 0.069) for the difference in the completion of diagnostic follow-up, favouring the CHW intervention arm. An ER of 1.00 was found.

A study of primarily Hispanic women also showed positive results. Statistically significant (*p* = 0.005) higher rates of endoscopy appointments at three months were found in the CHW intervention arm compared to the usual care arm [21]. Significantly (*p* = 0.019) higher rates were also found for the completion of endoscopy at six months. A trend towards significance (*p* = 0.086) was found for completing faecal occult blood tests (FOBT) after three months: a greater proportion of women in the CHW intervention arm completed the FOBT. No statistically significant differences in the completion of endoscopy at three months were found. The ER for this study was 0.75.

A third study with Hispanic women did not show statistically significant differences in the number of women returning for a second annual preventive exam compared to women receiving only postcard reminders [22]. Consequently, the ER was 0.00.

The mean ER for the outcome category “access to care” was 0.58.

### Health behaviour

Two studies showed statistically significant positive changes in behaviours related to cardiovascular disease risk for Hispanics in the CHW arm [19,23]. One study showed that the probability of achieving a high degree of improvement in eating habits relative to no change was greater for women in the CHW intervention arm than for women in the usual care arm (*p* < 0.001) [19]. Regarding physical activity, the probability of achieving a high degree of improvement in physical activity was also greater in the CHW intervention arm (*p* < 0.001). No statistically significant differences were found for smoking. The ER for this study was 0.43.

The second study reported statistically significant changes in salt intake (*p* < 0.001), cholesterol and fat intake (*p* = 0.01), weight control practices (*p* = 0.01), perceived benefits (*p* = 0.01), and perceived susceptibility (*p* = 0.01) in favour of the CHW intervention compared with the intervention based on providing basic educational materials [23]. No significant differences were recorded for perceived severity and self-efficacy. We found an ER of 0.71 for this study.

A third study focussed on physical activity within a Hispanic population [18]. Significant positive changes in the readiness to engage in vigorous physical activity and take up new physical activity were reported in the CHW arm compared to the usual care arm. Significant changes in moderate as well as vigorous physical activity were found, favouring the CHW intervention arm. No significant differences were reported for other physical behaviours (performing daily activities more briskly and incorporating physical activity into daily activity). The ER for this study was 0.67.

In a study on reducing diabetes risk factors among African Americans, none of the behaviours (dietary risk score, leisure-time physical activity, or BMI) showed significant differences [24]. Therefore, the ER was 0.00.

The mean ER for the outcome category “health behaviour” was 0.45.

### Health status

Two studies focussed on reducing cardiovascular disease (CVD) risk factors for Hispanics. One study showed a statistically significant (*p* < 0.001) change in diastolic blood pressure in the CHW arm compared to those receiving basic educational materials [23]. Trends towards significance were found for waist circumference (*p* = 0.09), non-HDL cholesterol (*p* = 0.10), and HbA1c (*p* = 0.09), favouring the CHW intervention arm. No significant differences were reported for BMI, weight, blood glucose or three other cholesterol measures. The ER for this study was 0.29.

The second study reported a statistically significant higher reduction in systolic blood pressure in the CHW arm compared to the usual care arm [19]. A trend towards significance (*p* = 0.051) was found for 10-year CHD risk. The other clinical measures (including BMI, cholesterol, and two other blood pressure measures) did not show significant changes. We found an ER of 0.22 for this study.

In a study on reducing diabetes risk factors among African Americans, no statistically significant differences were found for HbA1c or five other clinical measures [24]. As a result, the ER was 0.00.

The mean ER for the outcome category “health outcomes” was 0.17.

## Discussion

The present evidence for the effectiveness of CHW programmes for older adults from ethnic minorities is not univocal. In two studies, we found no significant differences [22,24]. In five studies, we found some positive effects. We did not find negative effects in any of the studies. Therefore, we conclude with some caution that there are indications that CHWs can help to improve health care use, health behaviour, and health outcomes among ethnic minority older adults.

The strength of the evidence differed by outcome category. Substantial effects on access to care (mean ER = 0.58) and on health behaviour (mean ER = 0.45) were found. The mean ER for health outcomes was considerably lower (mean ER = 0.17). This might be because an improvement in health status occurs as a consequence of an improvement in health behaviour and access to care initiated by CHWs. Therefore, effects on health outcomes in contrast to access to care and health behaviour are expected to emerge in the long term.

The quality of the included studies was considered to be sufficient, but not optimal. The strength of the effects found for access, health behaviour, and health outcomes did not seem to be associated with study quality.

CHWs may be less effective in improving health behaviour and access to care in older adults compared to younger adults belonging to ethnic minorities. A previous review [14] that focussed on ethnic minority adult women of all ages showed a higher percentage of studies that reported at least one positive result for access to care (100% vs. 66.7% compared to our review) and health behaviour (83.3% vs. 75%). This comparison should, however, be viewed with some caution given the differences in health focus and the type of studies for some of the included studies. Nevertheless, we consider the results of CHWs for older adults to be promising.

### Limitations

Our review revealed a number of methodological issues regarding the seven included studies. First, interventions were quite clearly described, and most details were reported except for training, supervision (content and duration) and CHW characteristics (e.g., age, gender, educational level, and payment). Therefore, we could not determine whether the type of training, supervision and type of CHW influenced the effectiveness of CHWs. A second issue was that several studies did not mention which components of the CHW interventions were effective and produced the reported effects. Third, we identified only a small number of studies. Therefore, robust conclusions on effectiveness could not be drawn. In addition, all studies have been conducted in the US which makes generalisation of the results of these studies to other Western countries questionable. Finally, none of the included studies reported outcomes regarding knowledge. We would expect that an improvement in behaviour through CHW counselling and education could also have improved knowledge. This hypothesis was confirmed in a previous review of studies of ethnic minority women of all ages in the US, in which an increase in knowledge was shown as a result of CHW education [14].

### Recommendations

First, further research is needed to draw more solid conclusions regarding CHW’s effects on the access to health care services, health behaviour, and health outcomes of ethnic minority older adults. Additionally, further studies should include knowledge as an outcome to examine whether CHWs can improve knowledge in ethnic minority older adults. For outcomes in which CHW interventions showed benefits, further research is needed to understand which components make the interventions effective in older adults belonging to ethnic minorities. This can be done by process evaluation whereby participants of the intervention are involved (providers of health care facilities, ethnic minority older adults, and CHWs). More uniformity in the interventions can be useful to determine which elements made the interventions effective. This also means that future studies should evaluate whether the type of training, supervision, type of CHW, and the function(s) of CHWs have an impact on the effectiveness of CHWs. Therefore, a clear description of the training and supervision procedures used and a specification of the functions and the type of CHWs that delivered the intervention are needed. In addition, an additional cost-effectiveness study would be useful to determine whether CHW interventions are a cost-effective alternative to health interventions to promote and prevent diseases.

## Conclusion

The implementation of CHW programmes might be effective in improving the health care access, health behaviour, and, to a lesser extent, health outcomes of older adults belonging to ethnic minorities, but further research is required to draw more solid conclusions. Further research is particularly important for Western countries in which the number of ethnic minority older adults has increased significantly because their health status is mostly unfavourable and their utilisation of health services is reported to lag behind that of the majority population.

## Additional file

Additional file 1: Table S1. General characteristics and outcome effectiveness of the included studies.
