# Supplementary material for: Community health worker interventions to improve access to health care services for older adults from ethnic minorities: a systematic review
Source: BMC Health Serv Res. 2014 Nov 13;14:497. doi: 10.1186/s12913-014-0497-1 (PMC4241213; doi:10.1186/s12913-014-0497-1)
Supplement: Additional file 1: Table S1. — General characteristics and outcome effectiveness of the included studies. [file 12913_2014_497_MOESM1_ESM.doc]

| **Table S2. General characteristics and outcome effectiveness of the included studies** | | | | | | | |
| --- | --- | --- | --- | --- | --- | --- | --- |
| **Author**  **Publication year** | **Study features**  Objective  Setting  Study design  Length of follow-up  Health focus | **Number (N)**  **Groups**  Eligible (N)  Enrolled (N)  Randomised (N)  Completers (N)  Groups  Interventions  Group (N) | **CHW**  Term used for CHW  CHW’s role  Paid or volunteer  CHWs (N)  Training  Supervision | **Target population**  Age (mean, SD)  Gender (% female)  Ethnicity/Race (%) | **Results** | **Effect ratio^1^** | **Mean effect ratio^2^** |
| **ACCESS** |  |  |  |  |  |  | 0.58 ((0.00+0.75+1.00)/3) |
| Hunter et al.  2004 | To test the effectiveness of a promotora programme to increase compliance with annual preventive exams among uninsured Hispanic women aged 40 and older living at the U.S.-Mexico border  US  RCT  NR  Chronic diseases | N=151  N=103  N=101  N=98  G1: promotora  G2: postcard  G1: postcard reminders and were visited by a promotora 2 weeks after the postcard had been mailed. Promotora facilitated appointment (re)scheduling  G2: received postcards in the mail 2 weeks before the month their annual exams were due  G1: N=51  G2: N=50 | Promotora  Case management  NR  NR  Training in intervention implementation—content and duration NR  NR | 50.3 (±7.5)  G1: 51.1 (±7.9)  G2: 49.6 (±7.1)  100%  96% Hispanic  G1: 100%  G2: 92.2% | Returned to the clinic for a second comprehensive annual exam  G1: 65%  G2: 48%  RR: 1.35 (95% CI 0.95-1.92) | 0.00 (0/1) |  |
| Jandorf et al.  2005 | To determine  whether a patient navigator would enhance CRC screening | N=125  NR  N=78  N=78 | Patient navigator  Case management, data collection, education,  outreach  NR  N=1  NR  NR | 61.2 (±7.8)  G1: 61.1 (±7.2)  G2: 61.3 (±8.4) | Completed FOBT after 3 months  G1: 42.1%  G2: 25.0%  *p*=0.086 | 0.75 (3/4) |  |
|  | participation beyond that due to physician recommendation alone  in a neighbourhood health clinic  US  RCT  6 months  Colorectal cancer | G1: patient  navigator  G2: usual care  G1: patient navigated. Patient navigator assisted with completing the screening process  G2: no patient-navigated services  G1: N=38  G2: N=40 |  | 74.4%  G1: 76.3%  G2: 72.5%  82.1% Hispanic  G1: 78.9%  G2: 85.0% | Had endoscopy appointment at 3 months  G1: 18.4%  G2: 0%  *p*=0.005  Completed endoscopy at 3 months  G1: 15.8%  G2: 5.0%  *p*=0.115  Completed endoscopy at 6 months  G1: 23.7%  G2: 5.0%  *p*=0.019 |  |  |
| Maxwell et al.  2010 | To test a peer navigation programme to increase adherence to diagnostic follow-up test after breast cancer screening among Asian American women  US  RCT  6 months  Breast cancer | N=176  N=176  N=176  N=116  G1: peer navigation  G2: usual care  G1: peer navigator conducted reminder phone calls, provided assistance to overcome barriers to follow-up—including emotional support, translation, proving information  G2: up to 2 phone calls by the Cancer Detection Programme case | Peer navigators  Case management  NR  N=1  3.5 day training including breast health, cancer, screening, study protocol, information about medical facilities  Meeting with Korean American physician once a week—monitoring activities, answering questions | 52 (±8.0)  100%  100% Korean American | Completed diagnostic follow-up after breast cancer screening (full case analysis)  G1: 97%  G2: 67%  *p*<0.001  Completion diagnostic follow-up after breast cancer screening (intention-to-treat analysis)  G1: 61%  G2: 46%  *p*=0.069 | 1.00 (1/1) |  |
|  |  | manager and a registered letter urging them to make a follow-up exam appointment  G1: N=92  G2: N=84 |  |  |  |  |  |
| **BEHAVIOUR** |  |  |  |  |  |  | 0.45 ((0.00+0.71+0.43+0.67)/4) |
| Gary et al.  2003 | To determine whether multifaceted, culturally sensitive primary care-based behavioural intervention implemented by a nurse case manager (NCM) and/or a CHW improves HbA1c and other diabetic control indicators in urban African Americans with type 2 diabetes  US  RCT  2 years  Diabetes type 2 | N=822  N=342  N=186  N=149  G1: NCM/CHW  G2: CHW  G3: NCM  G4: usual care  G1: combined NCM plus CHW—goal was 3 visits per year with each  G2: CHW intervention—45 to 60 minute face-to-face home visits and/or telephone contacts Preventive care—scheduling appointments and visits, providing education. Goal was 3 visits per year  G3: RN (certified diabetes educator), 45-minute face-to-face clinic visits and/or telephone contacts, direct patient care, | Community health worker  Case management, education  NR  1  NR  NR | 59 (±9)  G1: 60 (±7)  G2: 59 (±9)  G3: 59 (±11)  G4: 57 (±8)  77%  G1: 78%  G2: 78%  G3: 76%  G4: 74%  100%  African American | No significant  differences between CHW intervention and other groups in  dietary risk score, leisure-time physical activity index, BMI | 0.00 (0/3) |  |
|  |  | management,  education, counselling, follow-up, referral, physician feedback—goal was 3 visits per year  G4: continued on-going care from own health professionals and a newsletter on diabetic-related health topics  G1: N=36  G2: N=41  G3: N=38  G4: N=34 |  |  |  |  |  |
| Balcázar et al.  2010 | To promote behaviour changes to decrease  CVD risk factors in a high-risk Hispanic  border population  US  RCT  4 months  Cardiovascular disease | N=568  N=NR  N=328  N=284  G1: promotora intervention  G2: basic educational materials  G1: 8 health classes conducted by promotores—every week for 2 months. Follow-up—3 phone calls and small group sessions discussing changes and encouraging further changes  G2: basic educational | Promotores de salud  Education  NR  N=3  1-week training (16-18 hours)—content NR  NR | G1: 53.5 (±13.4)  G2: 54.0 (±13.2)  G1: 75%  G2: 68%  100% Hispanic | Adjusted post-intervention differences at follow-up  Salt intake  G1: 2.0 (±0.5)  G2: 1.8 (±0.5)  *p*<0.001  Cholesterol and fat intake  G1: 1.9 (±0.7)  G2: 1.7 (±0.6)  *p*=0.01  Weight control practices  G1: 2.0 (±0.6)  G2: 1.9 (±0.6)  *p*=0.01 | 0.71 (5/7) |  |
|  |  | materials provided in person at baseline  G1: N=192  G2: N=136 |  |  | Perceived benefits  G1: 3.7 (±0.4)  G2: 3.6 (±0.5)  *p*=0.01  Perceived susceptibility  G1: 3.5 (±0.4)  G2: 3.4 (±0.5)  *p*=0.01  Self-efficacy and perceived severity were not significantly different between the two intervention groups |  |  |
| Hayashi et al.  2010 | To evaluate the short-term impact of a lifestyle intervention on CVD risk factors and health behaviours among underserved middle-aged Hispanic women with one or more CVD risk factors  US  RCT  12 ± 2.5 months  Cardiovascular disease | N=1093  N=1093  N=1093  N=869  G1: lifestyle intervention delivered by CHWs  G2: usual clinical care  G1: 3 face-to-face sessions of assessment and counselling for nutritional and physical activity  G2: educational pamphlets on high blood pressure and high cholesterol, educational classes or verbal education  G1: N=552  G2: N=541 | CHWs  Education, data collection  NR  N=8  2.5-day hands-on training on conducting the study— trained by professionals specialised in lifestyle counselling, evaluation and cardiovascular health  Supervised by clinical staff member (RN)—content NR | G1: 51.8 (±6.4)  G2: 52.1 (±6.4)  100%  100% Hispanic American | High improvement in eating habits vs. no change  Relative risk ratio: 3.32 (*p*<0.001)  Low improvement in eating habits vs. no change  Relative risk ratio: 1.56 (*p*=0.105)  Worse vs. no change in eating habits  Relative risk ratio: 0.90 (*p*=0.681)  High improvement in physical activity vs. no change  Relative risk ratio: 2.11 (*p*<0.001) | 0.43 (3/7) |  |
|  |  |  |  |  | Low improvement in physical activity vs. no change  Relative risk ratio: 2.26 (*p*=0.006)  Worse vs. no change in physical activity  Relative risk ratio: 0.83 (*p*=0.507)  No significant between-groups differences for smoking |  |  |
| Coleman et al.  2012 | To assess the effect of a CHW programme on low-income Latinas’ readiness to change physical activity and on physical activity behaviours  US  RCT  12 months  Cardiovascular disease | N=1093  N=1093  N=1093  N=868  G1: lifestyle intervention delivered by CHWs  G2: usual clinical care  G1: 3 individually tailored  one-on-one counselling sessions —50 minutes each  G2: usual care for elevated blood pressure or cholesterol, healthy behaviour education, healthy lifestyle hand outs, referral to healthy lifestyle education classes | CHWs  Education, data collection  Paid  N=8  2.5 day on intervention delivery—trained by programme staff and other state programme partners on study protocols, nutrition and physical activity behaviour change, counselling, data collection  NR | 52 (±6)  G1: 52 (±6)  G2: 52 (±6)  100%  100% Latina | Readiness to engage in vigorous physical activity  G1: OR 2.34 (95% CI 1.77-3.09)  G2: OR 1.26 (95% CI 0.96-1.65)  Significant between-groups differences  Take up new physical activity  G1: OR 4.53 (95% CI 3.37-6.10)  G2: OR 2.22 (95% CI 1.68-2.95)  Significant between-groups differences  Perform daily activities more briskly  G1: OR 4.52 (95% CI 3.39-6.02) | 0.67 (4/6) |  |
|  |  | G1: 552  G2: 541 |  |  | G2: OR 2.72 (95%  CI 2.06-3.59)  No significant between-groups differences  Incorporate physical activity into daily activity  G1: OR 5.21 (95% CI 3.82-7.09)  G2: OR 3.06 (95% CI 2.30-4.08)  No significant between-groups differences  Moderate physical activity  G1: OR 2.19 (95% CI 1.57-3.07)  G2: OR 1.10 (95% CI 0.80-1.50)  Significant between-groups differences  Vigorous physical activity  G1: OR 3.37 (95% CI 2.38-4.77)  G2: OR 1.11 (95% CI 0.77-1.59)  Significant between-groups differences |  |  |
| **HEALTH OUTCOMES** |  |  |  |  |  |  | 0.17 ((0.00+0.29+0.22)/3) |
| Gary et al.  2003 | To determine whether | N=822  N=342 | Community health worker | 59 (±9)  G1: 60 (±7) | No significant differences | 0.0 (0/6) |  |
|  | multifaceted, culturally sensitive primary care-based behavioural intervention implemented by a nurse case manager (NCM) and/or a CHW improves HbA1c and other diabetic control indicators in urban African Americans with type 2 diabetes  US  RCT  2 years  Diabetes type 2 | N=186  N=149  G1: NCM/CHW  G2: CHW  G3: NCM  G4: usual care  G1: combined NCM plus CHW—goal was 3 visits per year with each  G2: CHW intervention—45- to 60-minute face-to-face home visits and/or telephone contacts. Preventive care—scheduling appointments and visits, providing education. Goal was 3 visits per year  G3: RN (certified diabetes educator), 45-minute face-to-face clinic visits and/or telephone contacts, direct patient care, management, education, counselling, follow-up, referral, physician feedback—goal was 3 visits per year  G4: continued ongoing care from own health  professionals and | Case management, education  NR  1  NR  NR | G2: 59 (±9)  G3: 59 (±11)  G4: 57 (±8)  77%  G1: 78%  G2: 78%  G3: 76%  G4: 74%  100%  African American | between the CHW intervention group and other groups for HbA1c, LDL cholesterol,  HDL cholesterol,  systolic blood pressure, diastolic blood pressure, triglycerides |  |  |
|  |  | a newsletter on  diabetic-related  health topics  G1: N=36  G2: N=41  G3: N=38  G4: N=34 |  |  |  |  |  |
| Balcázar et al.  2010 | To promote behaviour changes to decrease CVD risk factors in a  high-risk Hispanic border population  US  RCT  4 months  Cardiovascular disease | N=568  N=NR  N=328  N=284  G1: promotora intervention  G2: basic educational materials  G1: 8 health classes conducted by promotores—every week for 2 months. Follow-up—3 phone calls and small group sessions discussing changes and encouraging further changes  G2: basic educational materials provided in person at baseline  G1: N=192  G2: N=136 | Promotores de salud  Education  NR  N=3  1-week training (16-18 hours)—content NR  NR | G1: 53.5 (±13.4)  G2: 54.0 (±13.2)  G1: 75%  G2: 68%  100% Hispanic | Adjusted post-intervention differences at follow-up  Diastolic blood pressure (mm Hg)  G1: 79.8 (±9.3)  G2: 75.5 (±10.6)  *p*<0.001  HbA1c (%)  G1: 6.5 (±1.4)  G2: 6.6 (±1.4)  *p*=0.09  Non-HDL cholesterol (mg/dL)  G1: 146.3 (±42.3)  G2: 152.4 (±43.4)  *p*=0.10  Waist circumference (in)  G1: 40.4 (±5.7)  G2: 41.0 (±5.9)  *p*=0.09  Other clinical measures (BMI, weight, Framingham risk score, metabolic syndrome, LDL cholesterol, HDL  cholesterol, total cholesterol, triglyceride level, fasting blood glucose, systolic blood pressure) were not significantly different between the two intervention groups | 0.29 (4/14) |  |

| Hayashi et al.  2010 | To evaluate the short-term impact of a lifestyle intervention on CVD risk factors and health behaviours among underserved middle-aged Hispanic women with one or more CVD risk factors  US  RCT  12 ± 2.5 months  Cardiovascular disease | N=1093  N=1093  N=1093  N=869  G1: lifestyle intervention delivered by CHWs  G2: usual clinical care  G1: 3 face-to-face sessions of assessment and counselling for nutritional and physical activity  G2: educational pamphlets on high blood pressure and high cholesterol, educational classes or verbal education  G1: N=552  G2: N=541 | CHWs  Education, data collection  NR  N=8  2.5-day hands-on training on conducting the study— trained by professionals specialised in lifestyle counselling, evaluation and cardiovascular health  Supervised by clinical staff member (RN)—content NR | G1: 51.8 (±6.4)  G2: 52.1 (±6.4)  100%  100% Hispanic American | Systolic blood pressure change  (mm Hg)  G1: -5.9  G2: -3.7  *p*=0.038  10-year CHD risk  G1: -0.009  G2: -0.005  *p*=0.051  No significant between-group differences for other clinical measures  (blood pressure ≥140/90 mm Hg, diastolic blood pressure, total cholesterol, HDL-C, ratio of HDL/total cholesterol, total cholesterol≥240 mg/dL, BMI) | 0.22 (2/9) |  |
| --- | --- | --- | --- | --- | --- | --- | --- |

^1^ The effect ratio (ER) was calculated by dividing the total number of (trending to) significant outcomes by the total number of measured outcomes. The ER ranged from 0 to 1. A score of 0 indicated that there was no significant effect, and a score of 1 indicated that all measured outcomes were significant.

^2^ The mean ER was calculated by summing the ERs for each outcome category and dividing the total score by the total number of ERs for that particular category.
